# The Non-Panel Movement

**Published:** 1913-11-29

**Authors:** 


### THE NON-PANEL MOVEMENT.

### Electoral Anomalies Under the Insurance Act.

THE remarkable extent of the Commissioners' powers under the Insurance Act is well exemplified in the recently published Regulations for the election of committees of doctors and chemists. To one clause in particular attention may be drawn. We refer to Clause 23, which runs:

(1) Where, owing to a deficiency of nominations or the failure of the persons entitled to take part in an election, or of any of them to exercise their rights of voting, it appears to the Commissioners that a substantial miscarriage of the election has occurred, the Commissioners may, if they think fit, declare the election to be void, either in respect of all the persons who are highest on the poll, or of such of those persons as they may decide.

(2) Where the election is declared to be void in respect of all the persons elected, the Commissioners shall order a new election to be held in the manner provided in these Regulations; and where the election is declared to be void in respect of some only of the persons elected, the Commissioners shall make such provision for filling the remaining vacancies as they think fit.

In a letter to the *Times* of November 17, Dr. Percy Rose, in quoting this clause, says with justice: "From this it would appear that an election having been carried out right up to the counting of votes and placing of candidates, such election can be invalidated by the mere opinion of the Insurance Commissioners. The wording of the section actually gives the Commissioners ground for such action if one elector fails to vote. Other phrases in the clause make the matter worse by empowering the Commissioners to choose between elected candidates and to sanction the election of some while arbitrarily rejecting others. To the best of my knowledge such a method of controlling an election is entirely foreign to our islands."

### BUREAUCRACY AND AUTOCRACY.

The Act, of course, has given the Commissioners a power as nearly absolute as they could wish, for by Clause 65 ". . . any regulations so made shall be laid before both Houses of Parliament as soon as may be after they are made, and shall have effect as if enacted in this Act: Provided that, if an address is presented to his Majesty by either House of Parliament within the next subsequent twenty-one days on which that House has sat next after any such regulation is laid before it, praying that the regulation may be annulled, his Majesty in Council may annul the regulation, and it shall henceforth be void, but without prejudice to the validity of anything previously done thereunder." Very little reflection is required to show that the presence of Clause 65 disposes of the idea that the panel doctor, or anyone else for that matter, has under the Act any rights worthy of the name. Rights apparently conferred by the Act are liable to be nullified in effect at any moment by regulations issued by the Commissioners. Had the Insurance Bill received anything like proper discussion in the House it is safe to say Clause 65 would have had a good deal of attention before its insertion was allowed. But the guillotine dominated the situation, and "not one of the seventy-seven clauses of Part 1* of the Bill was open to consideration, although there were over 700 amendments down on the paper. Out of these 700 amendments some 470 were Government amendments, and they were inserted in the Bill without a word of discussion." ("The Real Lloyd George," by G. E. Raine.)

### THE B.M.A. NON-PANEL COMMITTEE.

The recently appointed Non-Panel Committee of the British Medical Association held its first meeting on November 19. It is of interest to note that beyond arranging matters relating to the organisation of the future work of the Committee the business done amounted to the registering of two protests: one directed against the wording of the notice to insured persons to select a doctor (Form 99 I.C.); the other protest dealt with the impending allocation of insured persons who have not chosen a panel doctor. Without any wish to disparage the efforts of the B.M.A. Non-Panel Committee, it is to be feared that with the best possible intentions its work will be nullified for the reason already advanced in these columns—namely, that the executive of the Association is a Council engaged in promoting the Act, and that panel and non-panel interests are incompatible on too many points for the same Association to be able to render effective service to both. For example, the most recent proposals of the Association are the raising of the annual subscription to two guineas, and the "establishment of a Special Voluntary Fund of four guineas per head for the thorough Organisation of the Profession." Non-panel members will be asking how the money subscribed to the special fund is to be expended, and whether perchance they may, if they subscribe, find they have been paying for the furtherance of schemes contrary to their own interests.

This remains to be seen; the claim of the Association to a capacity for both panel and non-panel interests will be tested when the Council has to decide whether or not it will act on some recommendation sent up by the Non-Panel Committee which clashes with the interests of the panel members, and it cannot be long before such a position arises if the new Committee acts up to the terms of reference. Meantime the registering of "protests" will do neither good nor harm. These furnish an expression of opinion and nothing more, and if addressed to the Commissioners will undoubtedly be transferred to the waste-paper basket. At the time of the recent resignations in the Wandsworth and other divisions of the B.M.A. the charge against the Council was that it constituted in practice, if not in theory, a pro-panel body engaged in working the Insurance Act. The resigning members claim that the charge has never been refuted, and so far as they may still be interested in the Association's policy they will rightly ignore mere expressions of opinion from

* National Health Insurance.

the Non-Panel Committee and look instead for some step by the executive showing both the inclination and the ability to further the non-panel members' interests.

The London Medical Committee, which is entirely non-panel in composition, has recently dealt with this question of the unallotted funds, and passed a resolution favouring the endeavour to obtain an injunction against the distribution of these funds by the London Insurance Committee. This is a step in the right direction, but there can be little doubt that this money will ultimately find its way, as an unearned bonus, into the pockets of the panel doctors. The Commissioners have intimated that they intend to get Parliamentary powers, if need be, to this end; they are more concerned to maintain the strength of the panel lists than to deal out justice to the insured public, whose compulsory contributions will to the extent of this unallotted money be expended in paying panel doctors for work which has been done by non-panel practitioners. The anxiety of the Commissioners to provide further remuneration (at the taxpayers' expense) indicates the fear of a falling away from the panel lists. Whether the present rate of payment will continue is more than doubtful if we notice what has happened in Germany, the country whose insurance system is admitted to have furnished the model for our own scheme. In a leading article in the *Times* of November 18 we read: "At first the German scheme worked well, but gradually the insurance committees or societies began to squeeze the doctors until they got the scale of payment down as low as 1d. a visit, even in so large and important a provincial capital as Breslau. The reply of the doctors was to combine in self-defence, which they did at first locally and spasmodically, with partial success. Conditions were improved and settlements made in some places, but no generally satisfactory arrangement was established, and the conflict has been continuously carried on. Now the new law, instead of bringing it to an end, has brought it to a graver and more general crisis than ever before. There is at present over the greater part of Germany something like open war between the insurance societies and the doctors, who have greatly extended and consolidated their organisation; and it is instructive to us to note that the heart of it is the Leipzig Union, because Leipzig has been held up as the great example of satisfactory arrangements and was the model on which our panel system was designed." Moreover "the Federation of Medical Associations, representing over 21,000 practitioners, passed with only four dissentients a strongly-worded resolution pledging themselves to renounce contract practice forthwith and until satisfactory terms are arranged, though they will continue to attend the sick."

Attention may again be drawn to the Conference of non-panel bodies to be held at 3 p.m. on Saturday, November 29, at the Waldorf Hotel, Strand. Any non-panel practitioner desiring particulars should communicate at once with the Secretary of the National Medical Union, 5 John Dalton Street, Manchester.

